# Feeling Valued and Adding Value: A Participatory Action Research Project on Co-creating Practices of Social Inclusion in Kindergartens and Communities

**DOI:** 10.3389/fpubh.2021.604796

**Published:** 2021-04-26

**Authors:** Dina von Heimburg, Susanne Vollan Langås, Borgunn Ytterhus

**Affiliations:** ^1^Faculty of Social Sciences, Nord University, Levanger, Norway; ^2^Department of Public Health and Nursing, Norwegian University of Science and Technology, Trondheim, Norway

**Keywords:** co-creation, health promotion, social inclusion, well-being, empowerment, social justice, participatory action research

## Abstract

**Background:** Contemporary public health problems connect to the social determinants of health, with a growing recognition of social inclusion as imperative to sustainable development. In this quest for social inclusion, early childhood and families are of particular interest. Although co-creation is suggested as viable path to support well-being, less is known how social inclusion might be co-created in practice. The aim of this study was to explore how Participatory Action Research (PAR) can be a tool for transformative practices in a local community, pointing to kindergartens as meeting places for recognizing social inclusion as a common value in early childhood.

**Methods:** A qualitative PAR study was embedded in a Norwegian municipality as an integrated part of their local public health work. The study involved a wide range of participants and stakeholders in three kindergartens and the wider community. Together, we explored potentials for co-creating social inclusion to achieve well-being through cycles of transformative actions and reflections. Reflexive thematic analysis was applied to generate patterns and themes in the data.

**Results:** The participants formulated and took on ownership to an inclusive agenda through the PAR-process. Acts of inclusion was framed by an intersection between political aims of achieving health and well-being for all and public value co-creation unfolding at the level of the place, in the context of the Norwegian welfare regime. To feel valued and adding value was seen as important aspects for social inclusion. Four themes were generated from analysis; (*1) Co-creating a shared vision of inclusive communities, (2) Becoming aware and empowered through caring, sharing and collaboration, (3) Places and spaces of inclusiveness in kindergartens and beyond, and (4) Valuing and practicing inclusion, and signs of transformative change*.

**Conclusions:** Through the PAR process, parents, kindergartens employees, community members and policy makers appear to have opened a creative toolbox for inclusive and transformational change through formulating and co-creating inclusion and well-being as public values. The results suggest that local actors might support adaptive social systems to taking on relational responsibility for inclusive processes and outcomes in the pursuit of well-being for all.

## Introduction

With a main focus on “leaving no one behind,” the historic and ambitious sustainable development goals (SDG) recognizes that societal development will only be sustainable if it is inclusive (1). Basically, this quest for inclusion is about human rights and human dignity toward health equity and well-being for all (2, 3). Studies shows that lack of social inclusion has severe consequences for individuals, relationships, organizations and communities, as well as the economy and society at large (4–8). Societies across the world still struggle to tackle complex public health problems (9, 10). Reaching the SDG's and promoting well-being for all depend on partnerships and co-creation across the whole of society, as stated in SDG # 17(1). However, reaching goals of inclusiveness and equity remains slow to progress, and transformative action is called for (2, 9, 11, 12). Especially, there is a call for action toward social inclusion in early childhood (13–16).

In the context of welfare, co-creation is described to alter the roles of citizens, users and professionals in ways that supports sustainable public value outcomes (17, 18). Although overall principles of co-creation are relatively well-worked out, there are surprisingly few long-term and comprehensive studies at the micro-level (19, 20). There is also a lack of knowledge on how co-creation processes might be inclusive and socially just (21, 22). This article explores how kindergartens as open social systems in interaction with place and space (e.g., social arenas, organizations, other institutions, and neighborhoods) might achieve common public values through participatory action research (PAR) in a Norwegian municipality. The study interweaves the fields of health promotion and co-creation.

Children are more likely to flourish when their families have the support they need, and where social networks and -conditions caters for health and well-being (5, 15, 23–25). The long-term beneficial effects of high-quality early childhood education is well-documented. It is good for everyone, but particularly beneficial for disadvantaged children (13, 14, 26). Family life is changing, alongside changes in community life, welfare systems and societies. Societal developments aligned with gender equality in work participation and a focus on high-quality education from an early age has accelerated kindergartens to become an important welfare institution in societies across the world (27). Co-creation is described as an approach to improve provision of welfare (17, 18, 28). A Swedish study found that parent engagement and involvement through co-creation enhanced the quality of the kindergartens (29). This study also suggest that parent involvement is not the norm in private and public kindergartens, pointing to a strong tradition of professionalism and passivation of citizens in the welfare state. Although this presumption is not empirically tested in Norwegian kindergartens, it is likely that these findings are transferable due to similarities within the Nordic welfare regime. Parents' engagement in their children's kindergarten values is also documented in Ytterhus' (30) study of Norwegian kindergartens as inclusive institutions for disabled children.

Parental and community engagement is increasingly seen as important to enhance healthy child development and learning (27, 31). According to OECD, countries face challenges related to lack of awareness and motivation from parents, lack of communication and outreach, parents' time constraints to being engaged, and increasing inequity and diversity among parents, with particular challenges associated with engaging ethnic minority parents (27). To address such issues, co-creation is seen as a promising approach (17, 29, 31). However, parent's involvement in kindergartens is still limited, or even restricted, both in Nordic countries and within OECD (27, 29).

By interweaving health promotion and co-creation, the current study builds on two basic premises: First, the objectives of the public sector is to create *public value*, situating the public as key actors in the construction of, and beneficiaries for public value creation (32–34). Second, the function of welfare states is to secure and support the *well-being* of its citizens (35, 36). Public health and well-being for all, leaving no one behind, is thus conceptualized as fundamental public values, with various measures to pursue this goal (37–39).

The view of social inclusion in the current study, builds on Prilleltensky's concept of mattering (7) as “to feel valued by, and to add value to, self, others, work and community” (p. 16). Thus, inclusion refers to results at micro-level, but is only reachable through processes at micro-, meso-, and macro- levels (7, 40). By conceptualizing social inclusion as a process, we rely on three distinct, but interlinked aspects, informed by critical theory; *social justice, relational responsibility*, and transforming *complex, adaptive systems*. These processual perspectives all relate to theoretical entries of transformative actions. First, processes to support social inclusion is viewed through the lens of social justice, coined as “participatory parity” (41, 42). The aspect of parity seeks to identify “*social arrangements that permit all (adult) members of society to interact with one another as peers*” (42) (p. 36). According to Fraser (41), participatory parity demands three distinct, but interlinked, pillars of justice; redistribution (typically economic in nature); recognition (typically cultural and relational in nature) and representation (typically political in nature). To Fraser (41), transformative processes relates to actions within all of these dimensions. Second, we view social inclusion as a process of relational responsibility (43). According to McNamee and Gergen (43) relational responsibility imply dialogical processes with two transformative functions; transforming the interlocuter's meaning-making of an action (e.g., acts of social inclusion and its consequences), and in altering the relationships between the conversational partners themselves. In such a social constructionist perspective, humans are conceived as relational beings (44). Meaning-making processes and the cultivation of inclusion relies on transformative dialogues and interactions where such processes bring people together into transformative and concerted action (45). In addition, we see social inclusion as a process unfolding in complex and adaptive social systems. Complex adaptive systems refer to systems that involve many components that adapt or learn as they interact, where the whole is more complex than its parts, where agents are interacting within a particular socio-ecological context, by adapting to each other's actions (46–49). Further, complex adaptive systems are approached as relationally constituted, where actors might create transformative actions with adaptive capacities in ecological systems (49). Such actions can trigger systemic transformative change, which refer to substantial changes in societal values, mindset, and behaviors (50).

The co-creation logic has recently gained traction within numerous governance areas and is described as a viable approach to tackling complexity aligned with unruly societal problems, and support citizen participation and public value creation in sustainable ways (17, 18, 34, 51–53). Co-creation is referred to as a promising approach to support health promotion, and tackle complexities inherit to health, well-being and equity (54, 55). A co-creation logic is linked to a “paradigmatic shift” in the public sector often referred to as “New Public Governance” (NPG), which is critical to the neo-liberal New Public Management (NPM) perspectives. In a NPM-dominated discourse, welfare is basically seen as a product that is “delivered” to the public/clients (36, 52, 53). While NPM give attention to service and cost-effectiveness, a co-creation logic directs the attention to collaboration, interactive networks, and bottom-up oriented forms of governance (17, 34, 55). The application of co-creation in this article is situated as an approach to pursue public value outcomes and thus embeds other “co-dimensions” such as, co-production and co-design. Co-creation is defined by Torfing et al. (52) as:

“*a process through which two or more public and private actors attempt to solve a shared problem, challenge, or task through a constructive exchange of different kinds of knowledge, resources, competences, and ideas that enhance the production of public value in terms of visions, plans, policies, strategies, regulatory frameworks, or services, either through a continuous improvement of outputs or outcomes or through innovative step-changes that transform the understanding of the problem or task at hand and lead to new ways of solving it*.” (p. 802).

Recently, advancing the perspectives described above, an approach to welfare coined as “relational welfare” has gained traction in Norway and beyond (36, 55, 56). The notion of “relational welfare” was initially coined by Hillary Cottam, privileging a radical attention on human relationships and relational responsibility (36). Basically, a relational approach to welfare make use of principles from co-creation to transform the relationship between the public and the welfare state, where inclusion and human dignity is key. By focusing on the settings of everyday life in communities, relational welfare connects to key pillars in health promotion (12, 55, 57). Relational welfare ties the concept of welfare to live well and flourish and nurture capabilities for doing so within acceptable structures. However, there is a need for research on how such a framework can be explored in practice.

This study addresses the need for more research on socially just micro-level co-creation, aligned with the need to accelerate health promotion practice. The purpose of this study is three-folded in exploring key elements in micro-level co-creation of inclusion and well-being in a kindergarten setting by focusing on: (1) how new roles might be played out, (2) how co-creation practices might look like, and (3) how public value outcomes might be successful at the micro-level. The research question is: *What are the processes and experiences parents, staff and local communities have in PAR when addressing social inclusion to support well-being?*

## Materials and Methods

### Methodology and Study Design

Based on the transformative purpose and the research question of the current study, PAR methodology was chosen as the research design. PAR is an approach to increase the possibilities for social transformation in specific contexts and situations, by involving stakeholders as active, participating subjects in the research process (58–60). The PAR-process brought together a wide range of stakeholders (see Table 1). Acknowledging the research process as a dialogical and relational processes, the study was theoretically based on a social constructionist theoretical stance (44, 56, 61). Accordingly, the PAR-process is conceptualized as a process of interactively co-constructing new knowledge and future-forming actions (62). This implies that PAR is seen as a collaborative, dynamic and abductive process, with ongoing conversations between theory, practice, relationally sensitive dialogues and self-reflections among all actors involved.

**Table 1:** Overview over the participants.

**Participants**	**Roles**	**Total *n***	**Within-group variation**
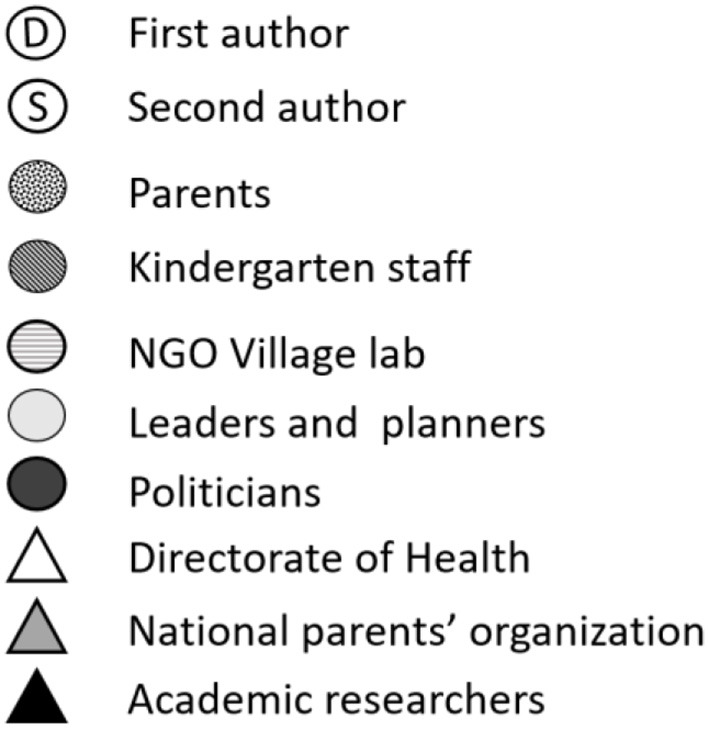	Parents i PFG	10	3 fathers, 7 mothers. Two migrants. 5 newly moved to Levanger. Two were out of work.
	Kindergarten staff in PFG	9	3 leaders, 3 kindergarten teachers, 3 assistants (1 man, 8 women).
	Participants in parents' meetings	105	90 parents, 15 staff
	Leaders and planners	5	1 executive leader, 2 sector leaders, 2 planners/coordinators
	Politicians	6	Members from the local council, representing 6 political parties. 2 men, 4 women.
	NGO Village labs	2	Representing two local communities in which the kindergartens are situated
	Outsider focus group (OFG)	6	Transdisciplinary representation of 4 academics, 1 participant from the Norwegian Directorate of Health, 1 participant from the Norwegian kindergarten parent's organization

### Study Context

The study was situated in a Norwegian municipality, where the first author of this article works as a public health coordinator. The second author has two different roles in this project. First, she participated as a parent and have generated data together with the other parents. Second, she is recruited as a co-researcher, because she gives voice to a group of citizens that very often are kept silent. She grew up in what she coins as an “outsider-society,” with lived experiences of social exclusion and bullying. The PAR revealed her relevant competencies and interests of academic work. Together with the third author and relevant stakeholders, they came together to nurture social inclusion and well-being for all as a shared public value, and mobilize joint action.

The Norwegian Public Health Act (63), adapted in 2012, was important for developing this study. This act explicitly embraces the social determinants and the “health in all policies” perspectives, and explicitly recognizes the role, responsibility of and accountability systems for the local governance level. Local governments are requested to promote participation and work knowledge-based to engage the local community in its developments aligned with a whole-of-society-approach. The study is based in a Norwegian mid-size municipality with ~20,000 inhabitants. Since 2014, this municipality had adopted public health and equity in health and well-being as main policy goals in their masterplan, where co-creation was a key strategy [see (64) for details of this policy process]. Thus, the current study is rooted in a local analysis of public health policy priorities in the municipality, in accordance with legislative demands.

To contextualize the study, a description of Norwegian kindergartens is required. In Norway, kindergartens have gone through radical changes during the last decades parallel to becoming a universal welfare institution. In 1975, childcare in Norwegian kindergartens was regulated by a legal Act (65). The kindergartens were organized under the Ministry of children and family affairs as a supplement to family caring. In 2006 there were a radical shift, which gave all children from the age of 1 year of age a legal right to kindergarten access. The responsibility for kindergartens were transferred from the Ministry of children and family affairs to the Ministry of education. They became an educational service and the first step into the public authorities' ambitions of lifelong learning. At date 92.2% of children enter kindergartens in Norway (66), and in the municipality participating in this study, 97.2% of the children are enrolled. Even though all Norwegian kindergartens are regulated by a common framework plan and national legislation (67), the majority of institutions are still private. Municipalities are local authorities for all kindergartens, regardless of organizational form, and are obliged to provide guidance and ensure that practices follow current rules and regulations. The children and the parents involvement are legally regulated to respectively, “be heard” and “participate,” e.g., through parents councils and in joint council committees (67). However, these regulations usually regulate that just a few of parents are active and involved.

### Participants, Data Sources, and Data Material

The current study involved three kindergartens in a Norwegian municipality, including parents/guardians, kindergarten staff, policy makers, boundary spanning coordinators/advisors, administrative leaders and local politicians from the municipal council. Parents in the kindergartens and kindergarten staff acted as a critical reference group in the study, whereas a strategic sampling of these actors formed a “participant focus group” (PFG) (68). A maximum variation strategy was applied to recruit research settings and contributors in the PFG (i.e., families: socioeconomic status, family structure, ethnicity, and gender; kindergartens: private and public, small and large, rural and urban; policy: across sectors). Selection of parents and staff was done by kindergarten leaders, where a recruitment procedure guided how they approached possible participants (i.e., to suggest participants based on the maximum variation criteria, focusing on people's regular roles as parents and employees, and not make suggestions based on previous engagements).

To ensure ethical issues of confidentiality and anonymity, an initial request to potential participants was forwarded to parents by the leaders of the kindergartens. Subsequently, a list of possible parents/guardians who agreed to be contacted was given to the first author, who contacted them for a written informed consent process. The data in the study consists from different data sources; individual interviews, three subsequent cycles of reflecting teams workshops (RT1-3), written notes, memos and closing reflection schemes from these RT-workshops, data from kindergartens and parents-meetings [including individually (anonymous) written evaluation from parents with closing reflections], and the researchers' diaries/memos at each cycle. In the PFG, we maintained a focus on parents and kindergarten staff, as they (by being significant adults in the kindergarten setting) are key stakeholders of inclusivity. In RT1 parents and staff participated, and in RT2, we included a wider range of relevant stakeholders in the municipality. Finally, to support reflections on transferability and academic novelty resulting from the research, we included an outsider focus group (OFG) of (transdisciplinary) researchers and policymakers at the national level to join our conversation in RT3. See Table 1 for an overview over research participants, and Table 2 for details of the data sources and processual and analytical procedures.

**Table 2:** Overview of the PAR process.

**Stage of the process, data generation and analysis**	**When was it done?**	**Why was this done?**	**How was this done?**
**Cycle 1:** Exploring the context and community inclusion ideals. 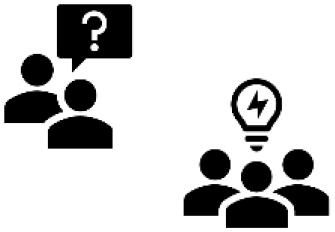 *Data*: audio recordings from 19 individual interviews (10 parents, 9 staff), researchers' diaries and memos.	Sept. 2017 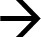 May 2019	*Preparing the context and participants:* Initial interviews with PFG served two main purposes: (1) negotiating meaning-making on inclusion through reflexive dialogues, and (2) preparing the actors for engaging in the research process and enhance trust. The conversations spurred the participants to talk about what they thought was important and allowing them to ask questions to the researcher.	A scoping review and theoretical frameworks were initially explored and used to prepare deliberative interviews (69) with PFG (parents and kindergarten staff), using a semi-structured guide as a conversational resource. The participants themselves chose the setting for the interview. In one of the interviews we used a professional translator. The interviews served as a stepping stone into the further process. The process was inspired by the BIKVA-approach to co-creation (70) but where our design was further developed to fit a dialogical and relational focus on transformative action.
**Cycle 2:** Discovery on common ideals and planning future-forming actions. 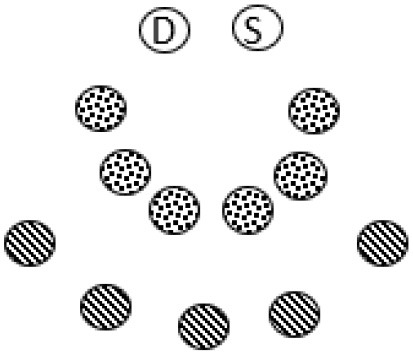 *Data*: video and audio from the RT, workshop notes (3 sheets), researchers' diaries and memos.*Thematic analysis:* step 1–3	May 2019 (RT1) 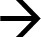 Sept 2019	*Reflecting team # 1 (RT1):* Engaging participants in the planning of future-forming actions. Negotiating a common dream, reflect on key issues/themes, and deliberate on possible steps to be taken. Disrupting dominant discourses between parents and staff, support reflection, dialogue, and preparedness to act.	Constructing a preliminary thematic analysis from the interviews, presenting and deliberating initial findings with parents and kindergartens staff through RT1 (step 1–3 in the thematic analysis). The RT1 process was inspired by Asset Based Community Development (71) and Appreciative inquiry (72). We asked questions like “How can we create stronger and more inclusive communities among families who have children in kindergarten?” and “Imagine five years ahead, what have we done together to achieve a common vision of inclusiveness?”
**Cycle 3:** Compiling actions in the kindergartens to improve inclusion. The Key Action was the Parents meetings. 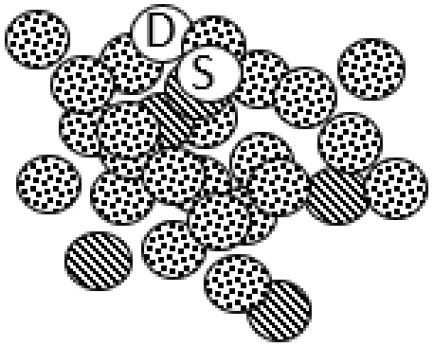 *Data*: Participatory observation, researchers' presentations, diaries and memos, workshop notes from parents (38 sheets), written evaluations from parents (90 forms), 3 memos from kindergarten staff, 3 memos from parents.	May 2019 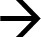 Nov 2019. Key actions: Sept. 2019	*Realizing and evaluating new actions:* Based on RT1, we ended up with zooming in on a key action – the parent meeting. This action became an important arena for constricting practices and data in the process, and to efficiently widen the circle of actively involved stakeholders beyond those participating in the PFG. The purpose of addressing the parents meeting was twofold: (1) to deliberate on the dream, raise awareness and empathy, and cultivating a we-culture of common concern and relational responsibility, (2) compile data from a wide range of critical stakeholders.	Author 1 and 2 collaborated with the PFG to plan and facilitate the parent's meetings. The dream and tentative themes from the initial analysis was consolidated with the participants, and we told stories of in/exclusion. The key event of the meetings was sessions of reflections in groups of parents, were also staff, to some extent, participated in the dialogue (inviting staff to join the conversation was requested by the parents themselves). They reflected on short narratives describing children's and parent's stories of being excluded and disvalued, which culminated in questions on how parents and staff could support acts of inclusion in the kindergartens and the wider community. At the end of the meeting, all of the parents individually filled out a written evaluation with closing reflections and suggestions for further actions.
**Cycle 4:** Reflecting on experiences, exploring implications. 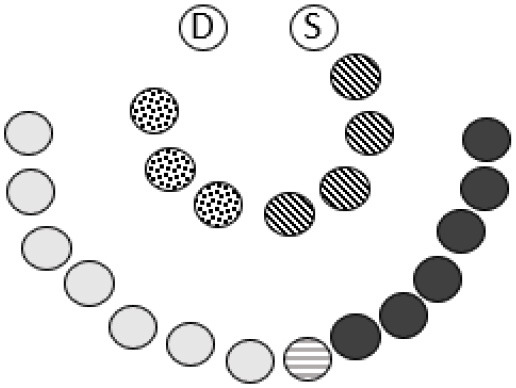 *Data:* Video and audio from the RT, Participatory observation, researchers' presentations, diaries and memos, workshop notes from participants (11 sheets), written closing reflections from participants (15 forms), 3 memos from kindergarten staff.	Nov 2019 (RT 2)	*Reflecting team # 2 (RT2):* Reflection and dialogue was facilitated to disrupt dominant discourses between kindergarten actors, other sectors, politicians and local NGO's as separate social systems. Reflect on what we had learned from the actions and suggest possible implications for policy development. Tinkering out suggestions on how a “we-culture” made up of inclusive acts might be enhanced.	Initially, the context for the research was explained and framed, and tentative findings from the research was presented by the PFG and reflected upon by the wider group of stakeholders. We posed questions like: “What have you experienced so far, and what are you hoping to happen next?” “In ten years, what has been done in the municipality to enable us to move closer to the vision?”, “what would you have been proud to transform?.” After deliberation, we organized the participants in groups (max variation of diverse stakeholders within the groups), to deliberate on how we can go on together to achieve the dream.
**Cycle 5:** Exploring co-impact. 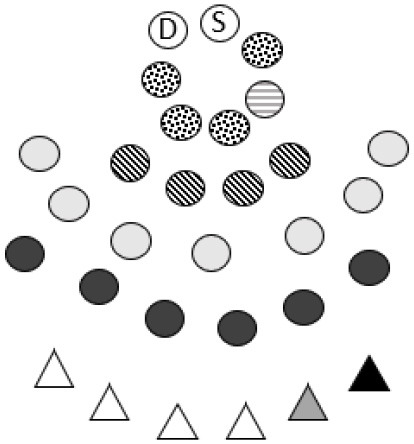 *Data:* Video and audio from the RT, Participatory observation, researchers' presentations, diaries and memos, workshop notes from participants (8 sheets), written closing reflections from participants (9 forms), 3 memos from kindergarten staff.*Thematic analysis:* step 4–6.	Nov 2019 (RT 3) 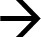 Subsequent analysis until August, 2020	*Reflecting team # 3 (RT3)*: Reflections on if, and how, the PAR-process has transformed conceptions of roles and actions in the quest for inclusion. Construct generative and reflexive dialogues toward transformative and sustainable change. Tinkering out what we can learn from the process, impact transferability of learning into other settings, and construct novel knowledge resulting from local experience and meaning-making.	Initially, tentative findings were presented and deliberated. All actors reflected upon what how the PAR-process had an impact on role identities and inclusive actions. We borrowed questions from Pearce (73), such as: (a) what are we making together? (b) how are we making it? (c) what are we becoming as we make this? And (d) How can we make better social worlds together? (p. 53). We examined what we had done and learned, asking questions like: “What might have transfer value to other settings beyond kindergartens, and other municipalities than ours?,” “what of these learnings can be important for national guidelines?,” “what is theoretically interesting?.” Subsequently, all authors analyzed the final dataset and revised the initial themes.

### Research Ethics

Formally, an ethical approval to conduct the study was granted by the Norwegian Social Science Data Services (NSD; project number 56952). Written informed consent was obtained after a full description of the study to the participants. There are two important ethical dilemmas that needs attention. First, there is always a risk for participants in action research in general (59, 74), and presumably in co-creation, that they felt obliged to satisfy the researcher heading and facilitating the group. This is especially relevant for participants with less formal power related to kindergarten and the local community. We tried to minimize such tensions by using RT-workshops (where participants could talk and reflect without being interrupted, and where we agreed on “rules” for inclusion and recognition). The first author prepared and engaged with participants to empower and support the parents. When addressing power asymmetry, some parents demonstrated a strong motivation to empower other parents in underprivileged social positions to participate, which is also documented by Dyregrov (75). Second, the researchers that participated in the generation of data (author 1 and 2) critically reflected on their own subjectivity at all stages such as avoiding any marginalization of the participants (76). When writing and reporting, all three authors aimed to do this in a respectfully manner toward all participants.

### Data Analysis

The data was analyzed through the use of reflexive thematic analysis (TA) (77, 78), following six steps: (1) *Familiarization with the data*, (2) *Coding the data by de-construction*, (3) *Generating initial themes by re-constructing the data material*, (4) *Reviewing themes*, (5) *Defining and naming themes*, and (6) *Writing up*. See Table 2 for details on the analytical procedure.

Important steps of reflecting together was organized as a series of three RT workshops (79). Step 1–3 in the reflexive TA, based on the initial interviews, resulted in seven preliminary themes which served as conversational resources in the succeeding circles of action and reflections (RT1 and the parent's meetings). The initial themes were: (1) *to be recognized and appreciated*, (2) *relationships and meeting-places which invites for participation*, (3) *diversity as a resource*, (4) *children as relationship- and community builders*, (5) *raising awareness and building culture for inclusion*, (6) *The kindergarten in the community, and the community in the kindergarten* and (7) *A common ground for upbringing and childhood are created by us right now*. After experimenting with inclusive actions in the PAR-process, a revised preliminary analysis was presented and negotiated in RT2 and 3. The entire dataset was finally analyzed by all authors. Data from interviews and RT workshops was initially audio-coded (80) and key sections were transcribed verbatim. Coding and thematizing data were supported by NVivo 12.

The two-stage review procedure in reflexive TA serves as an in-built quality mechanism for generating meaning and key themes, where the proposed themes are reviewed against the coded data and the entire dataset in a transparent manner (77, 78). The analytical procedure was recursive, moving back and forth between the different phases. The initial analysis of the individual interviews was performed by the first author, and then negotiated, reviewed and deepened throughout the research process. Throughout analytical process, a wide range of actors (see Table 2) reflected upon the research process, including the conditions affecting the situation of study, thinking interpretively about particular patterns aligned with reflexive engagement with the data. The internal validity of the results was enhanced by the second and third authors' discussions in the analytical process and writing the article together. The quality of the research was addressed through usefulness and “co-impact” (58, 59, 81).

Through the process of analysis, four main themes was generated to frame our results: (1) Co-creating a shared vision of inclusive communities, (2) Becoming aware and empowered through caring, sharing and collaboration, (3) Places and spaces of inclusiveness in kindergartens and beyond and (4) Valuing and practicing inclusion, and signs of transformative change. Table 3 provides an example of how theme 1 was generated by following the procedure described above.

**Table 3:**
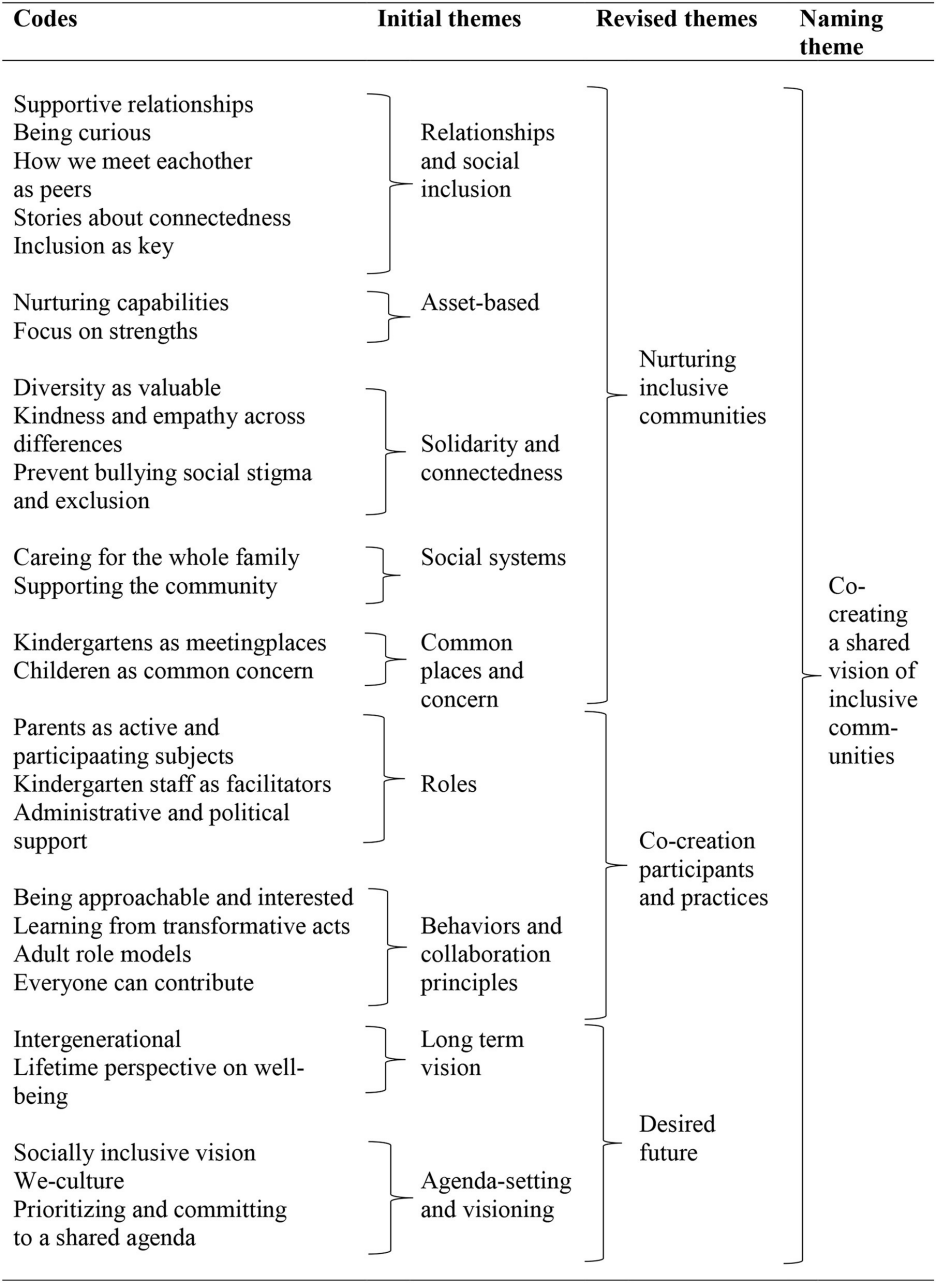
Examples of final analysis across the dataset - Theme 1 “Co-creating a shared vision of inclusive communities.”

## Results

Four main themes were generated as a “thematic story” responding to the research question: *What are the processes and experiences parents, staff and local communities have in PAR when addressing social inclusion to support well-being?*

### Co-creating a Shared Vision of Inclusive Communities

A shared vision served as a platform for co-creating actions to building a “we-culture” of social inclusion. Throughout the initial interviews with the PFG, grounded in “giving every child the best possible start in life,” a common vision was formulated and deliberated throughout the RT workshops:

“*We work together to create the childhood conditions we desire, for the benefit of all. Together, we have contributed to all children getting the best possible start in life, and that all children and adults feel seen and recognized as an equal and valuable participant in the local community.”* (written materials from RT1-3).

Although the PAR-process included a wide range of stakeholders, the participants did not express disagreements on the formulated vision (which did not change during the study). Instead they were more interested on how they could move on together to realize the vision through joint action. Further, they expressed that they wanted to feel socially connected, to be recognized and included, and to contribute positively to the lives of others. Even though, that they agreed on a shared vision, they acknowledged that it implied various changes in roles of the actors involved. For the parents, this involved taking on an active role. As one parent said: “*we must take on responsibility for our peers*” (RT3). For the kindergarten staff, a shared vision of inclusive communities entailed re-envisioning their professional mandate to facilitate co-creation in their kindergartens and local communities, and by approaching the parents as resourceful and motivated collaborators in pursuing the vision. Re-envisioning their professional roles also included to address inclusion and well-being of the whole family, beyond the kindergarten's opening hours.

Through the new practices that were developed through the PAR-process, both administrative staff and leaders across sectors reflected upon how such practices can be further developed and used in the municipality. For the politicians, getting knowledge about the new practices became important to humanize policymaking, legitimize co-creation practices, and contributing with new ideas. Developing kindergartens as meeting places and community-builders to co-create inclusion as a public value, was thus a desired aim for parents, as well as for kindergarten and administrative staff, policy makers and other stakeholders involved.

During the PAR-process, a wide range of participants acknowledged difficulties of being included in the community. As a NGO-representative pointed out in RT2: “*Our community is a bit closed*.” Participants said that it is not easy to get to know people in the community, especially if one moves there from other places (domestic and abroad). It was evident that social inclusion was an important value for all actors involved, not only for citizens struggling with marginalized and vulnerable living situations. The participants focused on pursuing inclusion, friendship, appreciative curiosity and mutual support as a main strategy to achieve well-being, including to prevent damaging relational patterns such as bullying, neglect, and abuse. They acknowledged the need for social inclusion of all and expressed a commitment to stop and prevent marginalization and exclusion. Especially, they addressed a need for taking on an intergenerational perspective in this pursuit, starting from pregnancy and lasting across the lifespan. One of the parents wrote in her reflections after RT2: “*there is a broad consensus that this* [the dream] *is important and should be a priority*.” Moving on to reflecting on potential impacts, she expressed that she “*expects change from ‘midwife to the grave', a structure made in interaction with those participating at any given time (i.e., the people)*.”

Aligned with a framing that placed parents, staff and other adults as responsible actors in co-creating social inclusion, the participants also stressed the fact that they all are role models for the community of children. An NGO representative said in RT3: “*to be a good role model. This is where it all begins*.” Aligned with this quote, a parent questioned: “*How can children learn that this* [inclusion] *is the natural thing to do, if we don't practice it in the community of adults?*” Another parent said that it is not inclusion when only those standing outside of community structures are put together as a group: “*Then, it is segregation*.”

The participants described that diversity in kindergartens (e.g., ethnicity, gender, social status, and disability) was as a resource to overcome social exclusion and marginalization. Instead diversity nurture transformative acts of tolerance, empathy and curiosity. For example, participants from the PFG told stories about how parents overcame anxiety and hostility toward minority families, as their own child became best friends with children from minority backgrounds. Other participants told stories of “otherness” and diversity as something that is genuinely interesting for children, pointing to that it is the adults, that bring forward conceptions of diversity as something “strange” or even “scary.” Valuing diversity, and at the same time, combating injustice through cultivating empathy and communities of common concern was important issues in the initial interviews and in the RT-workshops. Also, the participants generally expressed that diversity was important for open mindedness, learning and creativity. When reflecting on diversity and entanglement between social systems or groups in the kindergarten, a politician referred to observations of separate social systems in the community. In RT2, talking about the potentials of kindergartens as universal welfare settings, he said: “*I really like the idea of maybe creating some kind of a ‘mega-subculture' in kindergartens – 97%, you said? Then, in a way, everyone has a chance to form the social networks you are talking about.”* The participants shared stories about their motivation to engage in co-creation was enabled by a shared vision of creating an inclusive society. Thus, the vision became a common reference and an enabler for transformative action in their everyday life.

### Becoming Aware and Empowered Through Caring, Sharing, and Collaboration

Although the participants generally held some awareness of the importance of inclusion at the beginning of the PAR-process, many felt disempowered to act. Across the data, acts of caring, sharing and collaboration was valued as significant for transformative change. In the initial interviews, some of the participants said that people tended to be together, but still segregated in the kindergarten setting. One of the staff described that:

“*We see it at parenting meetings, those who know each other well, they come and sit down together. And then you have those who are always sitting alone. You can early notice who is on the “outside” in the kindergarten, both among the kids and the parents. They are probably also falling a bit “on the outside” of social life beyond kindergarten. There is something about finding a community outside the kindergarten as well*.”

One of the parents reflected on awareness and empowerment this way:

“*I believe that we need to open up our eyes a little more. We must create a culture where it is common and natural that we care about each other. It's not like inventing gunpowder, anyway. But of course, why haven't I thought about it earlier, to reach out to this person I do know who is, while he is sitting in the kindergarten and looking at his child, partly alone or alone. Why haven't I done anything about it? If more people are aware, and perhaps if the kindergarten makes us more aware of it, then maybe more people, who are in a well-functioning group outside the kindergarten, can go together to bring them along. It is easier to do this as group or a community*.”

Another parent participating in RT2 put it this way, reflecting on the need for joint action to achieve change:

“*This is a big job, right? And it's easy to think. Do I have the time for all this in my busy schedule? But then you have to bear in mind that, if I do a little, and you do a little, and you do a little* [pointing out in the room]. *Small things like, a little change, if everyone does it, then we are well on our way*.”

In RT3, one of the parents referred to small acts with potentially large impacts, such as “*giving a smile, despite being busy*.” Across the data, the participants reflected on transformative acts of inclusiveness as “contagious.” Paying inclusion forward was described as being aware and empowered to act, not only within the kindergarten setting, but by spreading into other social systems. One parent wrote in the evaluation form after the parent-meeting “*this was an important reminder. Social inclusion, recognizing every individual, and taking the time to do so. We are all important for this community.”* The participants agreed on this, and another parent suggested in RT2: “*The kindergartens, the way you have rigged it, has an impact on the parents as a group.”*

The parents also addressed key barriers and the need for tinkering out responses to tensions and dilemmas. For example, after conducting the first parent meeting, some parents said that the gap between norms and actual practices needed to be reflected upon to enable inclusive actions. In the closing reflections evaluation, reflecting on the impact of discussions on the presented narratives, one parent wrote: “*It was obvious what we should be saying. It is probably not quite so strait forward. Perhaps pose question to challenge us more, like ‘why don't you', not only ‘what you ought to do'?*.” This input, which came from the first parents meeting, made us revise the questions posed in the subsequent meetings. We experienced that posing such questions led the participants to reflect even more about their own role, and to how to overcome barriers to inclusive and transformative actions.

The parents described micro affirmations and recognition from other families and kindergarten staff as constitutive for their feeling of being a competent parent and being valued. Across roles, the participants recognized that every single person has capacities, abilities and gifts which can support to develop personal capabilities, as well as enhancing the capabilities of others. The parent's expressed that adding value to others, in the kindergarten and community, also added value to themselves. For example, parents with a refugee background referred to a sense of pride and recognition, when they were invited to cook traditional food from their own culture in the kindergarten or could teach the kids some words from their mother language. Others, who had construction skills, expressed that they felt valued by contributing to build the physical environment in the kindergartens.

The participants reflected upon that social inclusion in community life is best done by the community itself, albeit that the public authorities have the legal responsibility. Parents said that it was very important for them to be met by the kindergarten staff in a supportive and appreciative way. They also noted that being recognized by the children and parents in the kindergarten community “*gave a different kind of feeling than when the staff cared about me and the kids*” referring to that other parent's didn't have to do this as a paid job (researcher's memo). The parents said that recognition from children and other parents were constitutive for their feelings of being worthy and empowered. The participants talked about the importance of being met with respect and recognition. The participants enhanced the importance of meeting each other as peers, not as roles constrained with social status (e.g., approached as a doctor, cleaner, migrant, leader or a person with mental health problems). One parent said: “*I had great help from meeting other families on neutral ground in the kindergarten, so that I gradually became part of this community just by being present*” (researcher's memos). The parents said that they meet in kindergartens on equal ground; they take part in the setting because of their children. As some of the participants with lived experience of severe life difficulties reflected in the individual interviews and in the RT's; this is a radically different context than taking part in a welfare setting because you struggle with difficulties such as mental health problems, substance abuse or crime. Parents with lived experience of marginalization said, “*no one wants to be a charity case*” (researcher's memo), and instead emphasized the need to fulfill valuable social roles in the community. Generally, the participants talked about a desire to transform the discourse from being “vulnerable” to being “able,” with prospects of joining communities of support.

By intersecting visions of inclusion, awareness and joint action, the participants widened their repertoire. The vision was taken forward on the participant's own initiative. In one kindergarten, the parents' initiated events to create a community of mutual support. They highlighted the vision in their written invitation to the other parents. Throughout the study, the aspect of becoming aware of the importance of inclusion was a key issue. One planner said in RT2: “*What you have done in the kindergartens, it is about raising awareness, and what you are doing with us now, it is also raising awareness. And if you manage to find some ways to work like this in the whole local community. Then I believe one can get quite far* [referring to the vision].” Raising awareness *per se* was also linked to ways of doing it, where compassion and enthusiasm was coined as key issues. For example, the participants addressed that “*people who are engaged in a good cause is truly contagious, and what then is a better cause than our children?”* (researcher's memo). When reflecting on the learning from actions made through the study, one of the executive leaders in the municipality said in RT2: “*We know what we should be doing. But still, we don't do it. So, what you have done here, is to tackle this, in ways that has enabled us to talk about what is important, what really counts.”*

The participants also addressed that inclusion doesn't happen in a vacuum. One family lived in the refugee reception center (this story was referred to in RT2, and in individual interviews with parents and staff). A kindergarten staff talked to the mother when they planned the child's birthday party. They translated and forwarded an invitation to the other families in the group. Some of the parents expressed skepticism to come to the refugee center, but the staff gently nudged them to join the party. This gentle nudge made everyone participate. When the initial barrier was crossed, the party became a good experience for all, and especially for the birthday child and its family. The mother expressed the experience this way:

“*When I was about to enter the hall, I saw that it was completely full, and then I was very happy. What made me especially happy was that they did not think of me, they did not look at me as a refugee, living in an asylum reception center, they just came and looked at me like the rest of the community. It was very special, it was very touching.”* (initial interview)

After the experience of conducting the parent's meeting in a new, inclusive and participatory way, all three kindergartens wanted to continue with this new format. One kindergarten employee said in RT3: “*previously, we haven't really thought about the parent's meeting as a meeting for parents. Rather, it has been about sharing information from the kindergarten*.” Neither parents, nor staff wanted to return to the “old and traditional format.” Also, they wanted to continue the practice of strategically placing parents around tables in parent's meetings (e.g., by using the children's names as seating placement to avoid the parents of lumping together with others they already know well). Additionally, they wanted to strengthen an atmosphere for informal conversations, such as sharing a meal together where also the kids could join, and where the staff looked after the kids when the formal meeting began. In one of the kindergartens, the staff expressed in a written memo that the parents wanted a new network meeting, where the kindergarten initially provides some information, and then the parents divide into groups to discuss topics that they are interested in based on their own needs (e.g., screen use, sleeping habits, setting boundaries, creating common “rules” for creating inclusive cultures). By establishing new practices, the kindergartens transformed the parents-meeting as an arena for peer support and community building more than an arena for sharing information from the staff.

### Places and Spaces of Inclusiveness in Kindergartens and Beyond

The participants described several spaces and places for supporting inclusion in the kindergarten and beyond. Before entering the PAR-process, most participants related social inclusion to aspects of kindergartens as a welfare service and institution. When reflecting upon how social inclusion can be supported beyond the kindergarten opening hours, one of the staff described the following in the initial interview:

“*We can support the linkage of social relationships between families in a much, much better way than we do today. It is about taking relational responsibility outside of the kindergarten's opening hours. I believe that we are very good right in our own little “space.” But to lift our gaze, to see, to join forces, and to build community beyond the walls of the kindergarten. We've talked a lot about early intervention, but what is that exactly? The most important thing for the kids is to have empowered parents. This has got lot to do about the parents' mental health, and about their social relations.”*

By participating in the study the participants said that they became more aware and empowered to address inclusion inside of the kindergarten setting, but also to expand transformative acts of inclusion beyond its institutional fences.

Through the PAR-process the participants said that, although, they previously had arranged for places and spaces where families could meet within the kindergartens opening hours, they strengthened their efforts to create such arrangements in inclusive ways throughout the process (e.g., monthly gatherings such as eating breakfast or have coffee together, visits with grandparents, concerts etc.). Also, the kindergartens opened for other aspects of inclusive participation within opening hours. One kindergarten invited two of the mothers, who lived at an asylum reception center nearby, to work in the kindergarten 3 days a week on a voluntary basis based on written internship contracts. For the children, this meant extra adults in their setting, who could provide play, support and trust, as well as experiences of diversity and showing tolerance and recognition. For the staff, this meant extra support. For the mothers, this contributed to create a sense of purpose, meaningful activity, new relationships, and learning the Norwegian language. One of the kindergarten staff reflected in a written memo: “*This practice has worked well for all parties; it is a win-win situation*.” When talking about her experiences of the internship, one of the mothers said in RT3: “*It is very good for me. I have a negative result from my asylum application. So, I cannot go to school, I cannot work. I'm just sitting at home. It is very boring. Now, I'm better. When the children are giving me a hug… It just makes me happy*.”

The participants from the kindergartens came up with practical solutions to support families to get to know each other. In the children's wardrobe, some of them chose to hang up pictures of the child, with names not only of the child, but also their parents. Some parents came up with ideas of hanging up pictures of the parents too, to support familiarization. One of the parents said in RT3, learning the names of the children and their parents enables a feeling of “*being someone, not just anyone*.”

When suggesting how the inclusive vision could be realized, the participants talked about the roles and responsibilities of the kindergartens and its staff. One parent wrote this in the evaluation form after the parent's meeting: “*Kindergartens should be taking on a more active role. If they know of somebody who struggles/are excluded, so try to provide support. Connect parents to others and so on*.” This quote illustrates key messages from parents as well as employees. The participants said that the kindergarten staff know a lot about the parents and children, and they have follow-up conversations with parents on a regular basis (formal and informal). When families are in trouble and need additional support from e.g., child protection services or special educational support, the kindergartens often take part in the network of support. Parents that were interviewed who talked about experiences of needing extra support, firmly believed that the kindergartens should have a key role when families are in trouble. This view was also supported by kindergarten staff in the individual interviews as well as in the RT's. Furthermore, all actors recognized the children as a common concern, and that friendships between children could serve as a starting point for bringing parents and families together, and thereby enable reciprocal support. Here, both parents and employees highlighted the function of the kindergarten staff as key for acts of inclusion, for example by “*supporting to introduce parents whose children spend a lot of time together*” (written memo from one of the kindergartens), and at the same time facilitate that all of the children form friendships and participate in play. The kindergarten staff also talked about changes in the formal conversations with the parents (individual meeting with parents), where they started to ask new questions; “what do enjoy doing in your leisure time? Do you know what's going on in your local community and would you like more information? How to overcome barriers to participation, is additional support needed?” Such questions served the purpose of bridging families to participate in other social arenas in the community.

Altogether, the participants expressed a desire to use the kindergarten as a facility beyond opening hours. It was generally a place where parents and children felt safe and familiar, which also was free of charge. One of the parents said:

“*One of the other parents invited me and my kids to buy pizza as we left the kindergarten. I really wanted to answer “yes,” but on my bank account I had like 200 NOK, which was the last amount of money. I had for the next 5 days. Instead of sharing this information, I quickly replied that, “no, unfortunately, we don't have the time today.” If the kindergarten had been open as a playground that very afternoon, so anyone could gather for dinner, no one would have needed to know if I had money on my account, because I could have made the spaghetti I had planned for dinner anyways and taken it with me*.” (researcher's memos)

The parents in the PFG expressed a desire to meet other families in the kindergarten beyond opening hours in much the same way, suggesting meeting there to make dinner together, and for the children to play together in the afternoons and weekends. Across the process, they told stories about such initiatives. This need was also facilitated by the staff, where some of the kindergartens started to not only let the parents use the outdoor facilities after opening hours, but also letting them use the indoor facilities (lending out the keys to parent who took on responsibility for such events). Initiated by the parents in one of the kindergartens, they also arranged an evening for sharing things with each other, where everybody could bring stuff they no longer needed (e.g., toys, clothing, shoes and so on), and they could take home what they could use. They noted that such an arrangement had two purposes; to serve as a social arena, where parents could get to know each other, and to share things, which is good for both the social and natural environment. Another transformative act was that leaders in the municipality started to pay attention to how the built environment could facilitate the kindergarten to be more welcoming and inclusive. For example, when choosing amongst solutions for building another kindergarten in the municipality, aspects of openness and family-oriented practices, attending to the collective and wider community, was preferred.

The participants talked about internet and social media as important arenas. In the parents meeting, the parents also suggested new actions to pursue; to create digital platforms for communication and inviting each other to join activities (such as meeting on the playground or in outdoors in the kindergarten after opening hours). Such platforms also created sharing of a variety of support. In two of the kindergartens, the parents initiated a Facebook-group to keep in touch, share information and material goods, and invite each other to happenings. In the third kindergarten, the parents found another solution, as they believed that it would be difficult to keep track on all the parents, and also acknowledging that not all parents had a Facebook-profile. Instead, the staff and the parents created a list with contact information to all the families, so that they were able to connect.

Moreover, the participants talked about inclusion by bridging families to participate in the wider community. In the initial interviews, the participants in the PFG expressed that a practice to link families to community life (e.g., leisure, education, and work) was not mainstream. Practices attending to inclusion was mainly focused on the kindergarten as an institution, and not bridging participation and relationships into other social arenas. The parents, and especially those who had few relationships to count on, expressed a need for information on where they could meet and form relationships with other families. Throughout the initial interviews, the participants became aware of the multiple roles they have that could support participation and connectedness beyond the fences of kindergartens as an institution. One parent said in the initial individual interview: “*Although I am the leader of* [name of the NGO], *I haven't previously thought about the kindergarten as an arena for recruiting other parents*.”

Throughout the study, the participants became aware of the transformative possibilities to bridge participation from the kindergartens into other arenas. One of the parent's story (documented in the researcher's memos) is an example of this. In the kindergarten, this mother got to know other parents, that invited her to join other activities in the community. She was recruited as a member of a local NGO, which facilitated voluntary work that led her to join a chorus. In this chorus she got to know students at the university, that supported her application (on a special quota, since she didn't have the formal requirements) to take on music teacher studies. Now, she is on her way to her “dream job,” and her family is flourishing. Partly inspired by this narrative and similar stories of relational pathways to flourishing, the kindergartens (staff and parents walked alongside) took on new actions to bridge families into other social arenas in the community. In a written memo from one of the kindergartens, the staff wrote: “*The kindergarten writes a letter* [info letter to the parents] *every month - we now expand the letter by including everything that happens of activities for children in the community for the upcoming month*.” Another kindergarten supported this need with hanging up a board in the children's wardrobe where both staff and parents started to share information of what was going on in their community.

Another example of adding value and being valued outside of the kindergarten's fences, was a mutually beneficial relationship with one of the neighbors living close nearby one of the kindergartens. A senior man, who was very interested in music and played various instruments, had a huge collection of instruments in his home. Every year, he invited the kindergarten to visit, playing for the children and letting them try out his instruments. He also took on a role as Santa Clause in the kindergarten every Christmas. This man told the staff that meeting the children was important for his well-being, as he felt valued by and added value to the kindergarten community.

The participants also talked about the built environment as important for bridging possibilities for inclusion and participation. Situating kindergartens at the level of the place, it became apparent that the physical distance to other arenas and the neighborhoods where the families lived was a key factor. One mother who didn't have a car, said that “*it is a bit difficult to visit other families when they live far away*” (initial interview). Across the data, it became visible that closeness matter, not only between people, put also as a spatial dimension, where issues of transport and opportunities to meet others impact on the families' options to engage. It became apparent that when people know their community and the options for participation, they are also enabled to share information and welcoming “strangers” to arenas and settings such as sports facilities, libraries, organizations, schools and playgrounds nearby and so on. The participants also told stories about visiting such places, and where the children later had brought their parents along to these settings, acting as a guide. Furthermore, possibilities to cooperate with other welfare institutions such as nursing homes, providing mutual joy for children and senior citizens, was dependent on closeness within the place (an example provided from one of the kindergartens). Although some aspects of coordination and integration with other institutions and settings already was accounted for, the PAR-process enabled the participants to open a creative toolbox for social change at the level of the place. For example, the participants addressed a need for tools that enabled them to gain knowledge of available resources and options for participation in their communities. Subsequently, this provided arguments for implementing a digital platform to support sharing of information (this digital tool is currently being implemented in the municipality).

### Valuing and Practicing Inclusion, and Signs of Transformative Change

Talking about the value of social inclusion, and enabling people to become aware and empowered, was a recurring pattern in the data. In RT2 (after the PRG had presented and reflected on their experiences and learning from new practices and actions resulting from the process), one of the chief administrative leaders in the municipality reflected on what she had heard us talking about, and explicitly became aware of all the NPM-inspired argumentation in the system:

“*In everyday life, in the kindergarten and at home, we can get stuck by attending to our own busy schedules. I guess it is probably not conceptualized as ‘learning outcomes' in kindergartens, but there is so much going on. You have the annual plans, and the planning wheels, and that is probably what they use their time on in parents' meetings. So, we probably don't talk about what really is of importance, that is, how we meet and include each other. That is what you have opened for here. And maybe, in these meeting places, people commit to support each other, because of the ways the processes are designed, to include everybody in reflections on what really is important values for us to create. And then they feel a commitment toward others around them, which I believe is very important.”*

The participants acknowledged that we are “in it together” to create the society they wanted to live in. One of the kindergarten staff illustrated this with a trampoline metaphor in RT2:

“*If we imagine a trampoline, it has many strings attached around for it to bounce. If that trampoline is the child, and the strings are all of us in here in the community; it's child welfare, it's special pedagogic services, it's the kindergarten, it's all of us. If one string after another fails, then the trampoline will not work. But if all are intact, and all are cooperating in the interest of the child, then the child will be fine too*.”

This quote illustrates the acknowledgment of a transdisciplinary, multisectoral and whole-of-society approach in the pursuit for inclusion. This was also visible in the participants' dialogues, when talking about on their own multiple roles. They related to each other more as fellow citizens rather than on their formal roles, and greeted each other with curiosity, respect and empathy. For example, both managers and politicians in the process told stories from their family life and work life, attending to personal experiences of social inclusion and exclusion, and being valued by others. All actors agreed to promote the “we-can-do-it-together” feeling that was enhanced through the research, acknowledging that welfare is something we create together to support individual and collective well-being. One of the parents said RT3: “*we are each other's local environment*,” responding to a need for deepening co-creation.

In RT2 and 3, all the three kindergartens parents and staff said that transformative change was already happening. “*I believe that this has ripple effects, I am already experiencing a friendlier and more inclusive community.”* She further talked about how the process had affected her personally: “*I walk out of this room as a better me, with more thoughts and knowledge about the importance of the village*” (Staff in her closing comments/evaluation). Another example is from one of the parents, reflecting over his experiences on RT2: “*You become aware that much of the power* [for change], *lies among the parents. I was involved in* [the name of the kindergarten] *when you had it there* [the parents' meeting] *and to make them aware that in fact everyone is important for each other. And I agree, there has been more smiles and greetings since then.”* These quotes illustrate experiences from everyday life in face-to-face-encounters that made personal, relational and social change.

The participants said that creating a “we-culture” in kindergartens provides a platform for working together to support nurturing childhood conditions for all children throughout their childhoods and into adulthood. In RT3, one parent said: “*I believe what we have done really matters in the long run, as the children grow up, when they start at schooling, and in upper and secondary school*.” The NGO representative responded: “*If we now collaborate and create the conditions for nurturing childhood environments, it will have a huge impact on the society in the long run*.” When reflecting on learning and impact from the process, participants took on commitments to forward the agenda to other social systems in their communities. The participants also noted that ripple effects of pursuing transformative acts of social inclusion through co-creation was promising and created hope for the future. In RT3, one of the politicians said:

“*I'm thinking of the butterfly effect, the most exciting part is how this work creates something new outside the target group, like that someone has started to fill their leisure time with something meaningful, getting a job, friendship, further education and so on*.”

He is pointing to a wider range of impact than the children, but where such impact also transmit back to the children. Other aspects reported were about balancing a normative “push” to participate in, and initiate, inclusive activities on a regular basis, pointing to that too much push could lead to stress or resistance. Moreover, they emphasized the importance of face-to-face invitations, saying that it is easier to participate and join a group if you feel sincerely welcomed.

Overall, the participants and stakeholders involved initiated and participated to co-create new tools and their implementation. The participants also talked about the methods and tools used to support reflection and co-creation. One of the parents said in RT2: “*If you are going to move a culture, then the culture is not in the walls, it is in the people. Therefore, I believe that this* [action research] *is a methodology that can create movements, getting many actors on board*.”

The participants described that negotiating power-relations, language barriers and time-consuming aspects appeared as challenging throughout the PAR-process. Although, the parents expressed motivation to participate in co-creation, they were also concerned about balancing individual benefits and needs to adding value to the community. Across the study, the participants pointed to the importance of continuously focusing on social inclusion, in formal meetings and informal dialogues. One of the kindergarten staff shared in RT3:

“*This we-feeling… We weren't that aware of it before, but now, we get feedback both from staff, but especially from parents, that they connect to and feel this “we-ness.” I really feel good about it, because then it is a community, not us versus them or them versus us, but it's we, it is us. And that is something I really carry on with me from this process*.”

The participants said that creating inclusion together should not be “a one-time-happening” or a separate project. Rather, it should become a “*lifetime*,” “*intergenerational*,” and “*mainstream*” approach to transforming acts of social inclusion in the community as the participants agrees upon in RT2 and RT3.

## Discussion

The aim of this study was to explore *what are the processes and experiences parents, staff, and local communities have in PAR when addressing social inclusion to support well-being?*. Social inclusion was put on the agenda as the most important common public value. The results suggest that exploring kindergartens as open social systems in interaction with place and space became a promising platform to support social inclusion and well-being for families. The results advocate that parents, kindergartens employees and local communities are able and motivated to co-create practices and acts of social inclusion. Successful micro-level public value co-creation seems to have some crucial ingrediencies; negotiating a shared vision, active facilitation to empower participants (parents, staff, and wider community), and support coordinated and joint action at the level of the place by placing community first, supported by institutions who are held responsible for outcomes. Based on the results while attending to the purpose of the study (i.e., transform roles, practices and outcomes at the micro-level within a co-creational framework), we will organize the discussion around three key issues: (1) *Framing social inclusion as a relational and co-created public value*, (2) *Grounding social inclusion in social justice*, and (3) *Coordinated and integrated systems to support inclusion and well-being*.

### Framing Social Inclusion as a Relational and Co-created Public Value

Through the PAR-process, a co-constructed vision through dialogues and reflections acknowledged social inclusion as a shared public value. The results of this study suggest that social inclusion can be framed as relational processes and a co-created value that cannot merely be “delivered” as a transaction or service. Although this study cannot provide a full answer to how social inclusion as a welfare issue can be co-created, the results shed light on promising and future-forming possibilities for inclusive communities. This means that transforming relationships between the state and the people means to create a new interaction that puts more power in the hands of citizens, and emphasizing the public sector should “work with” rather than “doing to” their citizens (36, 82). Although, the dominant welfare discourse in Norway and internationally still connects welfare to “institutions,” “professions,” and “services” (18, 29, 36, 83), the results from the current study suggest that such a framing can be disrupted and altered by re-envisioning welfare and well-being as a common concern, governed by the public authorities.

The results indicate that the participants altered their role-perception throughout the process, where roles and functions to create social inclusion was about feeling valued and adding value to others; to feel included and to include others. In this way, the results support Prilleltensky's (7) studies on “mattering,” focusing on the importance of both “feeling valued” and “adding value” to others and the community. In the case of social inclusion, the relationship between individual and public value seems to be reciprocal and dynamic, where the dynamic nature of relies on meaning-making processes, relating to personal experience and visioning a desired future. At the micro-level in the kindergartens, parents seemed to transform their roles from passive receivers to active co-creators of public value. Importantly, the results suggest that motivation to co-create relied on pursuing visions they themselves found valuable. Moreover, our results highlight that awareness of, and empowerment to act spread from the kindergarten setting and into other social arenas in everyday life. For this to happen, the parents valued a close and reciprocal collaboration with kindergarten staff. For kindergarten staff, the results suggested that the co-creational endeavor implied taking on new roles as facilitators and bridge-spanners for building networks of collective support. The results shows that the staff can act as community “change agents”; to facilitate a shared vision between staff and families, support framing-capacity of inclusion as a co-created value, and actively create conditions to nurturing empathy and empowerment, relational responsibility and collective action beyond the kindergartens' institutional fences. Attending to the micro-level, the role of policymakers, administrative leaders and politicians also changed, where they first and foremost acted as fellow citizens. They listened, learned from, and participated in dialogue with parents and frontline staff, where they contributed with ideas to support further inclusive practices. Our results advise that the “kindergarten community” can lubricate the machinery of inclusion on the community, and to identify, connect, and mobilize people, assets and places for the common good through active facilitation. By this, the participants in our study acknowledged that many actors can contribute to social inclusion, welfare and we-ness as a content-component of welfare and well-being. The hybrid roles depicted here are in line with previously described enabling skills required by professional co-creators at the front line (84).

Our results suggest that social inclusion is a public value that depends on human interaction, and where co-creation might accelerate progress through transformative acts of inclusion. The results of this study propose that social inclusion in community life is best done in the community, by the community, where actors relate to each other as a community of peers rather than upfronting formal roles. Importantly, social inclusion was not only about presence, or allowance to take part. It also depends on being granted full recognition by others, where community integration is important too. Practical implications of these findings advise a need for integrating welfare institutions with community development, increase opportunities for people (parents) to define and actively take part of creating solutions, and support public servant's skills and capacity to co-create at the micro-level. Here, welfare systems serve the function to frame meaning-making dialogues on public value outcomes, facilitate co-creation and joint action, and fill in the gaps when extra support is needed. These suggestions do not advocate to leave the concept of kindergarten as institutions governed by regulation and criteria for service quality, but rather to renegotiate their mandate and practices as meeting places and community builders. Such an expansion of mandate is in line with health promotion templates of working with communities and settings of everyday life to support empowerment and joint action (12, 57).

### Grounding Social Inclusion in Social Justice

Our results indicate that kindergartens in Nordic welfare states have the potential to answering to all of Fraser's three dimensions of justice (i.e., redistribution, recognition, representation). However, the acts of social inclusion presented in this article are heavily skewed toward recognition. Although our results to some extent refers to elements of redistribution (for example universal access to kindergartens which caters for diversity and inclusion, acts of sharing material goods within the kindergarten community, and acts of opening doors for parents to participate in education and work-life), central aspects within the redistributive realm relies heavily on politics and representation. Here, the who's, what's and how's in policy making also relates to other aspects of recognition than those addressed in this article, requesting an ecosystem of capacity-building and inclusive representation in democratic processes to make transformative change.

Although our results provide arguments for reframing the welfare content and practices into a grammar of co-created social inclusion and well-being, our research does not provide an argument for welfare state retrenchment. The legal standard of welfare is in the Nordic welfare states are based on re-distribution of economic resources. It is the Nordic approach to welfare that furnishes for (almost) universal enrollment in Norwegian kindergartens. Based on our results, we support Raphael (37) and Esping-Andersen's (38, 39) arguments for pursuing the Nordic approach to welfare as a “gold standard” societal model for health promotion. Rather, the question to be deliberated is how welfare states facilitate action for all, maintain support from the growing middle-class, and mobilize citizens to take part in joint action, independent of social status.

Although our results support that co-creation in kindergarten fits well with new trends for *ad hoc*-volunteering (85), this could rise dilemmas in terms of justice. For example, when parents who are not allowed to work (e.g., asylum seekers or people on social benefits) enter kindergarten as volunteers, there can be a fine line between being valued and accumulate capabilities on one side and adding value as “unpaid staff” on the other, where freedom to earn money might be restricted by law. If kindergarten's incentives for including parents as volunteers are economic, and not built on relational responsibility, such inclusive practices at the micro-level could lead to widen inequities. Furthermore, our results suggest that if the “push” to participate is too hard, people might resist. An unintended consequence of a “participatory imperative” could be shaming and blaming, making the situation even worse for families in struggle. Thus, taking on relational responsibility also should involve to empathize, acknowledge participation as dynamic and fluid, and respecting the right not to participate, without being shamed (86). Based on our results, we recommend that practitioners and policymakers should critically reflect on such possible dark sides and unintended consequences before embarking on new co-creation practices.

Our results frame social inclusion as a common good, bridging fairness to universal well-being. We propose that entangling social inclusion to fairness and well-being can advance the fluid and complex relationship between the welfare state, the settings of everyday life, and community development. Such a reframing of justice implies consequences for policymaking as well as framing capacity in micro-level co-creation processes as mentioned above (8, 14). The results from this study support Heimburg and Ness' (55) arguments for paying attention to relationships as a fourth element to complement Fraser's three-dimensional approach to social justice, by advancing a relational approach to welfare toward well-being for all.

### Coordinated and Integrated Systems to Support Inclusion and Well-Being

The results emphasized that to support social inclusion and well-being, the systems should be coordinated and integrated. Our study demonstrates potentials for bringing a wide range of actors together to negotiate new meanings and joint visions and actions through dialogue. Despite arguments to engage parents more actively in early education, the empirical evidence on the contribution parents make is scarce (31). To our knowledge, a participatory whole-systems approach has previously not been studied in a kindergarten setting, and where the present study contributes to fill a gap. Following Andersen (87), a relationally coordinated, co-creation approach loosens up the intersection between public sector organizations and the function systems working in integrated manners to achieve public value. Our results suggest how established views of boundaries between kindergartens as institutions and the wider community can be blurred and relationally coordinated, linking kindergartens to a wider socio-ecological context. However, closeness, not only between people, but also amongst places and spaces seems to cater for coordinated and integrated acts of inclusion. Neighborhoods and the built environment affect how people interact with each other in ways that facilitate social contacts and strengthen social ties (5, 14). Thus, policy implications from our results suggest to physically situate kindergartens by prioritizing collaborative opportunities at the level of the place. Moreover, the results imply practical implications to look at procedures for enrollment, where closeness between families' homes and the kindergarten seems to matter for inclusion. Implementation of action needs to cut across traditional silos and facilitate integrated and coordinated actions to maximize co-benefits within the scope of inclusiveness, fairness and well-being. However, such practices are dependent on the wider conditions and structures for enacting upon inclusiveness (3, 8, 9, 23, 25). Our results show that the ecology of micro-level practices is affected by factors ranging from micro-encounters amongst people to being ecologically impacted by macro-level policy and culture. Based on our results, we propose that transformative micro-level practices can facilitate learning and change amongst in other parts of the system and levels of society, embedded in complex, adaptive systems.

## Limitations

Although we acknowledge PAR as a viable pathway to transformative change, there are several limitations to this study. First, this study had a focus on adult's transformative practices where the children themselves were only indirectly involved. The research could be deepened and strengthened by adding on children's own acts and perspectives. Second, is the democratic imbalance in knowledge and power between family members, researchers, practitioners, politicians and other stakeholders. Although we actively worked to make such imbalances transparent, and proportionately prepared actors to engage, aspects of authority could eventually be a barrier to parity in the process. One aspect is language barriers coupled with having asylum seeker status. This could put some participants in a challenging position in order to openly express honest opinions and critical reflections. Another aspect of “pleasing” in order to achieve a socially desired position could also apply to other participants. Although such aspects of power imbalances always are present in PAR, we worked systematically to make power-imbalances transparent, and had continuous dialogues on these matters to enhance reflexivity in the process. A wide range of actors participated in the analytical process, but it is the author's reflections and constructions who leads on to writing up this study. Thus, the results should be viewed through critical reflexive lenses, where the researcher's roles had influence on the processes as well as analytical process. Moreover, the first and second authors are employed in the municipality that is the setting for this study. Such an “insider-perspective” is constrained with pros and cons, and where self-reflexivity is important. In this study, the OFG acted as a “reflexive tool” to support a critical distance. During the research process, there were few critical comments from the participants, even though we actively invited criticism, both in the RT's and individual (anonymous) feedback loops. Moreover, the processual design was facilitating deliberation to achieve consensus more than exploring tensions (see Table 2). This can be a possible limitation because important input could be missed if participants did not feel comfortable to express critical reflections. We acknowledge that the transformative aims of this study colored the lens of the first and second author in conducting the research. The third author did not take part as an insider in the process, and thus contributes with critical distance in the research process. Moreover, a limitation could be the difficulty of distinguishing between what is practiced and what is believed to be ideal in the interviews and RT's. We also acknowledge limitations due to the number of actors involved in the PFG, and that other participant's might have brought in other stories and perspectives. However, this limitation was partly buffered by involving a wider range of stakeholders through the parent's meetings. Finally, one might question the usability of such context-bounded knowledge for future research and theorizing based on results. Even though these concerns can be addressed as a common treat to all qualitative research's validity, they are even more relevant in PAR. Our response here is our nuanced and thoroughly descriptions on PAR's different stages and how it was carried out, where the process itself and our results suggest transferable learning to other settings and research agendas and further theorizing co-creation of social inclusion (59, 81).

## Concluding Remarks

The results from this study points to a necessity of making significant actors aware and empowered to participate in co-creating acts of inclusion and well-being. The micro-level co-creation practices explored in this research propose that the traditional way of defining public institutions might, and should, be questioned, breaking down strict lines between the public and public institutions, and between sectors and professional disciplines. The results indicate that kindergartens as a setting, by involving multiple stakeholders, can create transformative change, even in a short time span. Moreover, not only is it possible, it also was desirable from the perspectives of all participants involved. Overall, the results indicate that public value outcomes can be successfully co-created at the micro-level. Kindergartens can become unique arenas to bolster social inclusion, with potential to contribute solving some of the most pressing public health problems today such as loneliness, mental health problems, abuse and marginalization. Finally, we acknowledge that the concept of inclusion is multidimensional in nature, and dependent on a wide range of actors and societal structures, horizontally and vertically. Maintaining participatory parity, relational responsibility and coordinated, transformative actions in complex adaptive systems relies on strategic planning, (organizational) capacity building and political leadership. We recommend that future inquiry should address such multi-level issues to make the aspiring co-created changes described at the micro-level by our results, truly transformative and sustainable. Ultimately, welfare systems should secure accountability systems to support the profound message of UN's SDG's of “leaving no one behind.” and continuously push forward an agenda of inclusion at the micro-level and beyond (9, 38, 88). The research reported here has focused in transformative actions, and not on evaluating effects. Future research should address possible (long term) effects of inclusive co-creation practices on the micro-level by using a wide range of methodology, and importantly also explore how such micro practices connects to processes and actors at the meso- and macro-level.

## Data Availability Statement

Relevant data is contained within the article. To protect the confidentiality of the participants, video and audio data will not be made available. Other requests to access the data should be directed to the corresponding author.

## Ethics Statement

The studies involving human participants were reviewed and approved by Norwegian Social Science Data Services (NSD; project number 56952). The participants provided their written informed consent to participate in this study.

## Author Contributions

DvH was leading the work on initiating, planning and facilitating the PAR process, documentation of data, and writing up the manuscript. SL contributed as a co-researcher throughout all stages of the process. DvH preformed the initial analysis, and where DvH, SL, and BY analyzed the final dataset. DvH and BY revised the manuscript. All authors approved the manuscript.

## Conflict of Interest

The authors declare that the research was conducted in the absence of any commercial or financial relationships that could be construed as a potential conflict of interest.
